# Xanthine Oxidase Inhibitory Activity and Thermostability of Cinnamaldehyde-Chemotype Leaf Oil of *Cinnamomum osmophloeum* Microencapsulated with β-Cyclodextrin

**DOI:** 10.3390/molecules23051107

**Published:** 2018-05-07

**Authors:** Chi-Ya Huang, Ting-Feng Yeh, Fu-Lan Hsu, Chun-Ya Lin, Shang-Tzen Chang, Hui-Ting Chang

**Affiliations:** 1 School of Forestry and Resource Conservation, National Taiwan University, Taipei 106, Taiwan; r99625047@ntu.edu.tw (C.-Y.H.); stfyeh@ntu.edu.tw (T.-F.Y.); aisiteru555@gmail.com (C.-Y.L.); peter@ntu.edu.tw (S.-T.C.); 2 Division of Forest Chemistry, Taiwan Forestry Research Institute, Council of Agriculture, Executive Yuan, Taipei 100, Taiwan; fulan40@gmail.com

**Keywords:** *trans-*cinnamaldehyde, *Cinnamomum osmophloeum*, β-cyclodextrin, microencapsulation, xanthine oxidase inhibitory activity

## Abstract

The xanthine oxidase inhibitory activity and thermostability of *Cinnamomum osmophloeum* leaf oil microencapsulated with β-cyclodextrin were evaluated in this study. The yield of leaf oil microcapsules was 86.3% using the optimal reaction conditions at the leaf oil to β-cyclodextrin ratio of 15:85 and ethanol to water ratio ranging from 1:3 to 1:5. Based on the FTIR analysis, the characteristic absorption bands of major constituent, *trans*-cinnamaldehyde, were confirmed in the spectra of leaf oil microcapsules. According to the dry-heat aging test, β-cyclodextrin was thermostable under the high temperature conditions, and it was beneficial to reduce the emission of *C. osmophloeum* leaf oil. Leaf oil microcapsules exhibited high xanthine oxidase inhibitory activity, with an IC_50_ value of 83.3 µg/mL. It is concluded that the lifetime of *C. osmophloeum* leaf oil can be effectively improved by microencapsulation, and leaf oil microcapsules possess superior xanthine oxidase inhibitory activity.

## 1. Introduction

Cinnamon plants (*Cinnamomum* spp., Lauraceae) are woody species native to South and Southeast Asia, commonly used as food flavors and folk medicinal plants. *Cinnamomum osmophloeum* Kanehira has long been used as a medicinal plant. Natural products obtained from cinnamon plants display versatile bioactivities, including antibacterial, antifungal, antioxidant, and anxiolytic effects, etc. [1,2,3,4,5].

Plant essential oils and extracts with antimicrobial, anti-inflammatory, analgesic, anxiolytic, and other properties are widely used for perfumes, food additives, pharmaceuticals, and so on [6,7]. Essential oils are rich in highly volatile simple aromatics, monoterpenoids and sesquiterpenoids. The highly volatile character of essential oils results in the quick release to the environment of their components. Thermal-induced oxidation and degradation of the essential oil also occur easily. These restrictions limit the lifetime and utilization of essential oil products. Microencapsulation of highly volatile leaf oils with proper shell materials can help improve their durability.

*C. osmophloeum* leaf oils from different sources are classified into several chemotypes, mainly including cinnamyl acetate-type, linalool-type, cinnamaldehyde-type, and the others types. Among these chemotypes of *C. osmophloeum* leaf oils, the cinnamaldehyde type leaf oil showed xanthine oxidase inhibitory activity and anti-hyperuricemia effects [1]. Xanthine oxidase is the key enzyme that catalyzes the oxidation of hypoxanthine/xanthine to produce uric acid; excess accumulation of uric acid leads to diseases such as gout and hyperuricemia. Cinnamaldehyde is a common antiallergic, anti-inflammatory, and antipyretic drug in traditional medicine. Cinnamaldehyde is approved as a Generally Recognized as Safe (GRAS) natural food additive by the United States Food and Drug Administration.

Common microcapsule shell materials include gum arabic, gelatin, cyclodextrins, sodium caseinate, cellulose derivatives, sodium alginate, chitosans, etc. [8,9,10]. Cyclodextrins, amphiphilic hollow polysaccharides, have been used as effective carriers for food and drug delivery in the food, pharmaceutical and biomedical fields [11,12,13,14]. β-Cyclodextrin is one of the most common microcapsule shell materials with advantages such as photostability and thermostability [6,15,16,17]. Many plant essential oils contain *p*-cymene, a monoterpenoid with highly volatile properties, possessing analgesic and anti-inflammation activities. Because the half-lifetime of *p*-cymene is short; it brings the problem for the storage and utilization. Quintans et al. [18] microencapsulated *p*-cymene by β-cyclodextrin to improve its stability and pharmacological activity. *trans*-Cinnamaldehyde, a major compound in cinnamon leaf oil, is classified as a phenylpropanoid and cinammic acid derivative in the classification of natural products. Microencapsulated bioactive plant essential oils with more functional properties can be applied in the food and pharmaceutical industries. Jiang et al. [19] investigated the combination of β-cyclodextrin and its derivatives with *C. loureirii* bark oil, which was rich in *trans*-cinnamaldehyde (69.6%). Among the three materials studied, which included β-cyclodextrin (β-CD), 2,6-di-*O*-methyl-β-CD (DM-β-CD), and 2-*O*-(2-hydroxypropyl)-β-CD (HP-β-CD), the inclusion ability of β-cyclodextrin was better than that of the other two derivatives, so in this study, β-cyclodextrin was selected as the microcapsule shell material.

The purposes of this study were to microencapsulate cinnamaldehyde-type leaf oil of *C. osmophloeum* with β-cyclodextrin and examine the properties of the resulting leaf oil microcapsules. It was expected that the preservation of *C. osmophloeum* leaf oil would improve. Xanthine oxidase inhibitory activity and thermostability of *C. osmophloeum* leaf oil microcapsules were investigated to confirm the properties of the leaf oil microcapsules.

## 2. Results and Discussion

### 2.1. Composition of Cinnamomum Osmophloeum Leaf Oil

**Figure 1:**
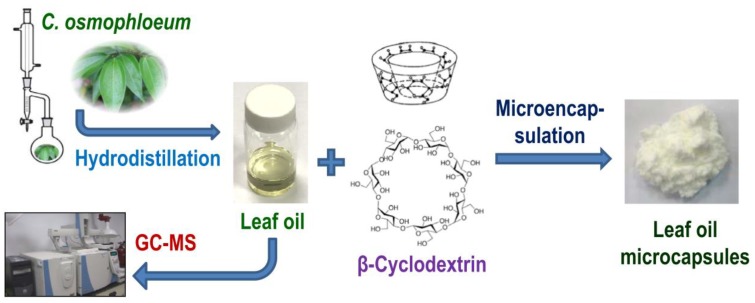
Schematic illustration of microencapsulation of *C. osmophloeum* leaf oil with β-cyclodextrin.

A schematic illustration of the hydrodistillation of *Cinnamomum osmophloeum* leaf oil and microencapsulation of the leaf oil with β-cyclodextrin is shown in Figure 1. 

**Figure 2:**
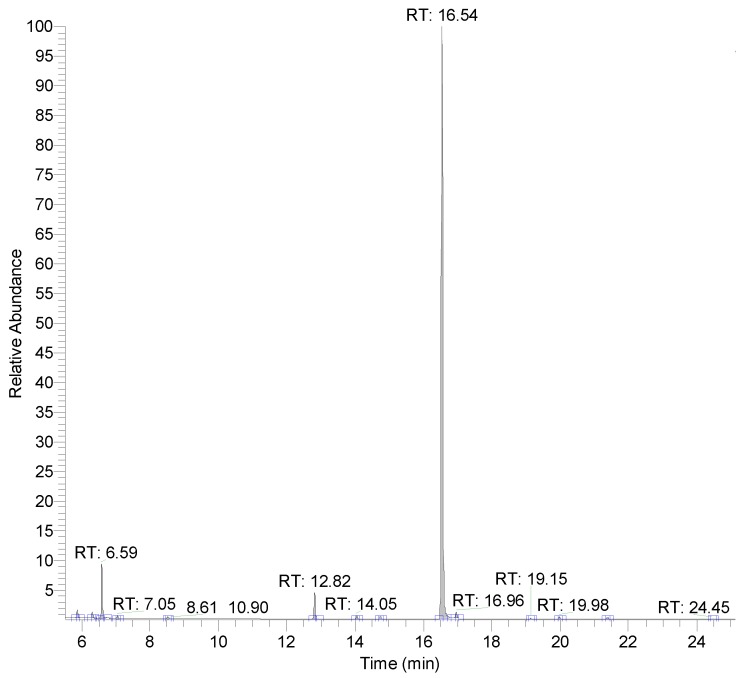
Gas chromatogram of cinnamaldehyde-type *C. osmophloeum* leaf oil.

The composition of the leaf oil was analyzed using GC-MS. Figure 2 shows a gas chromatogram of the leaf oil. The main constituents of the examined *C. osmophloeum* leaf oil were *trans*-cinnamaldehyde (84.13%), benzaldehyde (7.16%), 3-phenylpropionaldehyde (3.62%), α-pinene (1.09%), l-bornyl acetate (0.92%), and camphene (0.79%) (Table 1). The content of *trans*-cinnamaldehyde was much higher than that of the other constituents, so the leaf oil was classified as belonging to the cinnamaldehyde chemotype of *C. osmophloeum*. Based on the variation of major constituents, *C. osmophloeum* leaf oils can be classified into several chemotypes with individual bioactivity. Eugenol is a common compound found in the *Cinnamomum* plants. In this study, eugenol was a minor constituent (0.03%) of the selected leaf oil. The main constituents of the *C. osmophloeum* leaf oil were volatile aromatics and terpenoids.

### 2.2. Optimal Microencapsulation of C. Osmophloeum Leaf Oil with β-Cyclodextrin

The influence of the *trans*-cinnamaldehyde to β-cyclodextrin ratio on the microcapsule yield is shown in Table 2. β-Cyclodextrin completely dissolved in the solution (ethanol/water, 1:5 *v*/*v*) at 50 °C for 5 min, then the solution was cooled without adding the core material, *trans*-cinnamaldehyde, and no crystals occurred/precipitated even at 4 °C. The highest yield of microcapsules (83.7%) was achieved at the *trans*-cinnamaldehyde to β-cyclodextrin ratio of 15:85 (*w*/*w*), which was close to a 1:1 molar ratio. The other weight ratios significantly decreased the microcapsule yield ranged from 68.8 to 72.8%.

Microencapsulation of *C. zeylanicum* leaf oil and garlic (*Allium sativum*) oil in β-cyclodextrin was reported by Ayala-Zavala et al. [20]. The main components of *C. zeylanicum* leaf oil and garlic oil were eugenol (78%) and allyl disulfide (21%), respectively. The highest yields of *C. zeylanicum* leaf oil and garlic oil microencapsulation with β-cyclodextrin were obtained at the weight ratios of 16:84 and 12:88.

Another important factor in the microencapsulation was the ratio of ethanol to water in the reaction solution. For the 1:7 and 1:2 ratios of ethanol to water (*v*/*v*), yields of microcapsules were lower than that of the other ratios and below 80%. The 1:3 ratio of ethanol to water showed the maximum microcapsule yield (87.3%), but the difference was not statistically significant (*p* < 0.05) for the 1:4 and 1:5 ratios. With the optimal conditions for microencapsulation, the yield of cinnamaldehyde type leaf oil microcapsules was 86.3% (Table 2). The microcapsule yields were not statistically different between *trans*-cinnamaldehyde and cinnamaldehyde type leaf oil microencapsulated with β-cyclodextrin. The polarity of different ratios of ethanol to water in the reaction solution may influence the co-precipitation during microencapsulation.

Hill et al. [16] investigated the stability constants (Kc) of *trans*-cinnamaldehyde/β-cyclodextrin inclusion complexes at three different temperatures (25, 35, and 45 °C), the KCs of inclusion complexes were 28.47, 19.06, and 18.41 L/mol, respectively; the thermodynamic results revealed that *trans*-cinnamaldehyde/β-cyclodextrin inclusion complex formation was a typical lower energy reaction.

### 2.3. Evaluation of the Thermostability of Leaf Oil Microcapsules by Dry-Heat Aging Test

**Figure 3:**
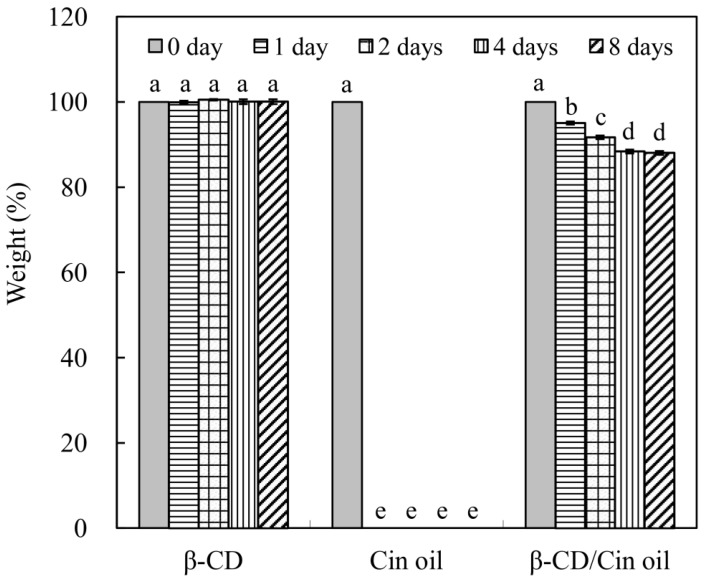
Changes in weight of leaf oil microcapsules during the dry-heat aging test. β-CD: β-cyclodextrin; Cin oil: *C. osmophloeum* leaf oil; β-CD/Cin oil: leaf oil microcapsules; Different letters (a–e) in the Figure refer to statistically significant difference at the level of *p* < 0.05 according to the Scheffe’s test.

The weights of β-cyclodextrin (β-CD), *C. osmophloeum* leaf oil (Cin oil), and leaf oil microcapsules (β-CD/Cin oil) were measured during a dry-heat aging test, performed at 105 °C in a ventilated oven, as shown in Figure 3. The weight percentage of β-cyclodextrin was kept constant during the accelerated aging test; β-cyclodextrin was thermostable at 105 °C. Trotta et al. reported that the decomposition temperature of β-cyclodextrin was ca. 250 °C according to the thermogravimetric analysis (TGA) [21]. The weight percentages of leaf oil were 11.7% and 0% after 30 min and 1 h of aging test, respectively (data not shown in Figure 3). Emission of *C. osmophloeum* leaf oil was quick in the high temperature external environment; high volatility of leaf oil was a problem for its utility. The weight loss of leaf oil microcapsules was 4.92% after 1 day of aging test. Comparing the weight loss between leaf oil and leaf oil microcapsules, it is clear that microencapsulation reduced the emission of leaf oil in the early stages of the dry-heat aging test. Weight percentages of leaf oil microcapsules were 88.4% and 88.1% after 4 and 8 days of the aging test. The weight percentages of leaf oil microcapsules after 4 days and 8 days were significantly lower than those before 2 days. This corresponded with the leaf oil content in microcapsules. Microencapsulation of leaf oil with β-cyclodextrin thus slowed down the volatility of the leaf oil, revealing that the thermostability of *C. osmophloeum* leaf oil was effectively improved by encapsulation with β-cyclodextrin.

**Figure 4:**
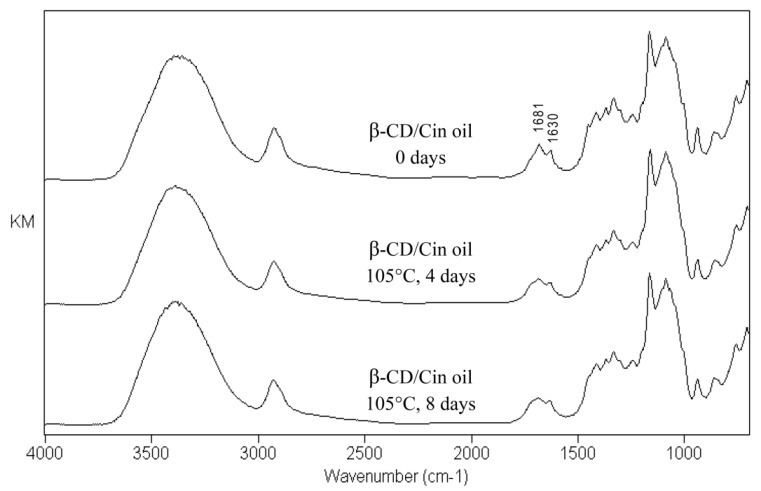
FTIR spectra of *Cinnamomum osmophloeum* leaf oil microcapsules after the dry-heat aging treatment.

Changes of the characteristic peaks occurred in the FTIR spectrum of *C. osmophloeum* leaf oil microcapsules (β-CD/Cin oil) after the dry-heat aging test, as shown in Figure 4. The relative intensities of the absorption peaks at 1630 cm^−1^ and 1681 cm^−1^ decreased during the aging period. Peaks at 1630 cm^−1^ (for C=C stretching vibrations) and 1681 cm^−1^ (for C=O stretching vibrations) are the characteristic absorptions of the extended conjugation structure of *trans*-cinnamaldehyde [22], the major constituent of leaf oil. The decrease of these absorption bands with the aging period resulted from the emission of *trans*-cinnamaldehyde in the environment at 105 °C. The characteristic absorption bands of β-cyclodextrin are 3386 cm^−1^ (for O-H stretching vibrations), 2926 cm^−1^ (for -CH_2_ asymmetrical stretching vibrations) and 1165 cm^−1^ (for C-O stretching vibrations) [23,24,25]. These characteristic peaks of β-cyclodextrin didn’t show any differences in the FTIR spectrum after the dry-heat aging test; revealing that the molecules of β-cyclodextrin were thermostable during 8 days of aging. The results from the FTIR spectroscopic analysis ensured the consistency of the thermostability of β-cyclodextrin observed in Figure 3. Most constituents of the leaf oil were control released from the microcapsules during the dry-heat aging test.

### 2.4. Xanthine Oxidase Inhibitory Activities of C. Osmophloeum Leaf Oil Microcapsules

Wang et al. [1] evaluated the xanthine oxidase inhibitory activities of ethanolic extract, hot water extract, and essential oil of cinnamaldehyde-type *C. osmophloeum* leaf; leaf oil, which contained 75.1% of *trans*-cinnamaldehyde, possessed the best activity with a half maximal inhibitory concentration (IC_50_) value of 16.3 µg/mL. In this study, *trans*-cinnamaldehyde content in examined leaf oil was 84.1% (Table 1), and the IC_50_ value of xanthine oxidase inhibitory activity of leaf oil was 11.2 µg/mL (Table 3). Xanthine oxidase inhibitory activity of *C. osmophloeum* leaf oil increased with the content of *trans*-cinnamaldehyde in leaf oil. 

Kamimura et al. [26] reported that antioxidant activity of carvacrol, the major constituent of oregano and thyme plants oils, microencapsulated in hydroxypropyl-β-cyclodextrin was lower than that of free carvacrol. Bioactivities of essential oil/constituents after microencapsulation might vary with the different core materials and coating materials [24,27,28,29]. No research about the xanthine oxidase inhibitory activities of *C. osmophloeum* leaf oil microencapsulation with β-cyclodextrin has been reported in the scientific research literature. The IC_50_ value of xanthine oxidase inhibitory activity of leaf oil microcapsules (β-CD/Cin oil) was 83.3 µg/mL (Table 3). According to the leaf oil content in microcapsules (14.9%), the calculated IC_50_ value of active leaf oil in microcapsules was 12.4 µg/mL, which was comparable to leaf oil alone. Results indicated that the xanthine oxidase inhibitory activity of *C. osmophloeum* leaf oil wasn't affected by microencapsulation with β-cyclodextrin. This is the first study to report the xanthine oxidase inhibitory activity of plant essential oil microcapsules. Cinnamaldehyde-type *C. osmophloeum* leaf oil microcapsules can be used as alternative natural inhibitor for hyperuricemia and gout.

## 3. Materials and Methods

### 3.1. Hydrodistillation of Leaf Oil

*C. osmophloeum* leaves were collected from the Fushan Research Center of Taiwan Forestry Research Institute (Taipei, Taiwan). Leaves (100 g) were hydrodistilled in a Clevenger-type apparatus (1 L) for 6 h to obtain to obtain the leaf essential oil. The yield of leaf oil was 0.82 ± 0.08% (mean ± standard deviation) calculated from duplicate tests.

### 3.2. GC-MS Analysis of Leaf Oil

The composition of the leaf oil was analyzed by using a Thermo Trace GC Ultra gas chromatograph and a Polaris Q MSD mass spectrometer (Thermo Fisher Scientific, Framingham, MA, USA) equipped with a DB-5MS capillary column (Crossbond 5% phenyl methylpolysiloxane, 30 m length × 0.25 mm i.d. × 0.25 µm film thickness). The oven temperature was initially held at 60 °C for 1 min, then increased to 220 °C at a rate of 4 °C/min and held for 2 min, and finally increased to 250 °C at a rate of 20 °C/min and held for 3 min. Carrier gas was helium at a flow rate of 1 mL/min, and the split ratio was 1:10. Components were identified by comparing their mass spectra (*m*/*z* 50–650 amu) with Wiley and NIST library data, Kovats index (KI) [30], and authentic standards of constituents. Quantification of components was obtained by integrating the peak area of the chromatogram by GC coupled to a flame ionization detector (FID).

### 3.3. Microencapsulation

Microencapsulation of leaf oil in β-cyclodextrin was carried out by a published co-precipitation method with minor revisions [20,23,31]. Based on the previous tests, higher yields were obtained by the co-precipitation method than with other microencapsulation methods. β-Cyclodextrin (C_42_H_70_O_35_, 5 g) was dissolved in ethanol/water solution (300 mL) at 50 °C for 5 min, then the solution was cooled down to 25 °C. Next leaf oil (0.88 g) was dissolved in ethanol (10 mL) and added to the β-cyclodextrin solution with stirring at 600 rpm for 1 h. The solution was refrigerated for 12 h at 4 °C; the precipitated microcapsules (complex crystals) were vacuum-filtered and washed with 50 mL of distilled water. Microcapsule powders were dried at 50 °C in an oven for 24 h until the powder weight remained constant. The yield of microcapsules was calculated using Equation (1). The leaf oil content in microcapsules was determined by the liquid-liquid partition method; the leaf oil was extracted by the ethyl acetate and isolated from the microcapsules.

Yield (%) = microcapsules (g)/[β-cyclodextrin (g) + leaf oil (g)] × 100(1)

The leaf oil content in microcapsules was determined by the liquid-liquid partition method; the leaf oil was extracted by the ethyl acetate and isolated from the microcapsules.

### 3.4. FTIR Analysis

Fourier transform infrared (FTIR) spectra of leaf oil microencapsulated with β-cyclodextrin were obtained using a FTS-40 spectrometer (Bio-Rad, Hercules, CA, USA) incorporating a Spectra Tech diffuse reflectance accessory unit. The spectral resolution is 4 cm^−1^, and the scanning range is 4000 to 400 cm^−1^ [24,32].

### 3.5. Dry-Heat Aging Test

A dry-heat treatment was used to evaluate the thermostability of specimens. Leaf oil microcapsules were subjected to accelerated aged using the standard dry-heat aging test (ISO 5630-1; CNS 12887-1) at 105 °C in a ventilated oven. Weights of specimens were measured during the dry-heat aging test for 1, 2, 4, and 8 days. After the aging test, chemical compositions of the leaf oil microcapsules were examined by FTIR analysis.

### 3.6. Xanthine Oxidase Inhibitory Assay

Xanthine is specifically oxidized by xanthine oxidase to form uric acid. The xanthine oxidase inhibitory activity was evaluated the ability of specimens, including β-cyclodextrin, leaf oil, and leaf oil microcapsules, to inhibit the formation of uric acid. The reaction mixture contained 117 µL of potassium phosphate buffer (50 mM, pH 7.8), 3 µL of sample (dissolved in DMSO) and 60 µL of xanthine oxidase solution (25 mU/mL). The reaction was initiated by adding 100 µL of 0.15 mM xanthine solution, incubated at 37 °C for 30 min, and stopped by adding 20 µL of 1 N HCl. The absorbance of uric acid was measured at 290 nm by an ELISA reader [33]. Allopurinol, a gout and hyperuricemia medicine, was used as a positive control. IC_50_ (half maximal inhibitory concentration) values are the concentrations of specimens required for the 50% inhibition.

### 3.7. Statistical Analysis

The results were presented as mean values and standard deviations. The data were grouped by the Scheffe test of the SAS software (version 9.2) at a level of significance of *p* < 0.05. The Scheffe test is a completely post-hoc multiple comparison analysis with stringent error control.

## 4. Conclusions

Constituents of *C. osmophloeum* leaf oil were identified by GC-MS and GC-FID. *trans*-Cinnamaldehyde (84.13%), benzaldehyde (7.16%), 3-phenylpropionaldehyde (3.62%), and α-pinene (1.09%) were the main constituents of the selected leaf oil. The optimal reaction conditions for microencapsulation were found at a *trans*-cinnamaldehyde to β-cyclodextrin ratio of 15:85 and ethanol to water ratios ranging from 1:3 to 1:5. The yield of leaf oil microcapsules was 86.3%. The characteristic absorption bands of *trans*-cinnamaldehyde were confirmed in the FTIR spectra of leaf oil microcapsules. The thermostability of leaf oil microcapsules were evaluated by the dry-heat aging test, and β-cyclodextrin was thermostable at 105 °C and had efficacy to retard the emission of *C. osmophloeum* leaf oil. The xanthine oxidase inhibitory activity of cinnamaldehyde-type *C. osmophloeum* leaf oil was not affected after microencapsulation with β-cyclodextrin and the IC_50_ value of leaf oil microcapsules in the xanthine oxidase inhibitory assay was in good agreement with the content of leaf oil in the microcapsules. These results revealed that cinnamaldehyde-type *C. osmophloeum* leaf oil microencapsulated with β-cyclodextrin possessed superior thermostability and xanthine oxidase inhibitory activity. Cinnamaldehyde-type *C. osmophloeum* leaf oil microencapsules have great potential to be used as a natural xanthine oxidase inhibitor for dietary supplements and treatment of hyperuricemia and gout.

## Figures and Tables

**Table 1:** Composition of *Cinnamomum osmophloeum* leaf oil.

Rt (min) *	KI **	Constituent	Formula	Content (%)	Identification Method
5.86	938	α-Pinene	C_10_H_16_	1.09	MS, KI
6.30	955	Camphene	C_10_H_16_	0.79	MS, KI
6.59	965	Benzaldehyde	C_7_H_6_O	7.16	MS, KI
7.05	982	β-Pinene	C_10_H_16_	0.36	MS, KI
8.61	1033	Limonene	C_10_H_16_	0.21	MS, KI
12.82	1164	3-Phenylpropionaldehyde	C_9_H_10_O	3.62	MS, KI
14.05	1198	Methyl chavicol	C_10_H_12_O	0.55	MS, KI
14.72	1219	*cis*-Cinnamaldehyde	C_9_H_8_O	0.50	MS, KI
16.54	1273	*trans*-Cinnamaldehyde	C_9_H_8_O	84.13	MS, KI
16.96	1285	l-Bornyl acetate	C_12_H_20_O_2_	0.92	MS, KI
19.15	1351	Eugenol	C_10_H_12_O_2_	0.03	MS, KI
19.98	1376	α-Copaene	C_15_H_24_	0.34	MS, KI
21.40	1420	β-Caryophyllene	C_15_H_24_	0.29	MS, KI
24.45	1517	δ-Cadinene	C_15_H_24_	0.02	MS, KI

* RT: Retention time (min); ** KI: Kovats index relative to *n*-alkanes (C9–C24) on a DB-5MS column.

**Table 2:** Yields of *trans*-cinnamaldehyde and leaf oil microencapsulated with β-cyclodextrin.

Specimen	Specimen:β-CD (*w*/*w*)	EtOH:H_2_O (*v*/*v*)	Yield (%)
*trans*-Cinnamaldehyde	0:100	1:5	0.0 ± 0.0 ^c^ *
	10:90	1:5	68.8 ± 1.1 ^b^
	15:85	1:5	83.7 ± 0.7 ^a^
	20:80	1:5	72.8 ± 1.2 ^b^
	25:75	1:5	70.8 ± 1.5 ^b^
*trans*-Cinnamaldehyde	15:85	1:7	75.5 ± 1.2 ^B^
	15:85	1:5	83.7 ± 0.7 ^A^
	15:85	1:4	85.0 ± 0.9 ^A^
	15:85	1:3	87.3 ± 1.1 ^A^
	15:85	1:2	65.0 ± 1.8 ^C^
Leaf oil	15:85	1:3	86.3 ± 2.0

* Different letters (a–c and A–C) in the table refer to statistically significant differences at the level of *p* < 0.05 according to Scheffe’s test.

**Table 3:** Xanthine oxidase inhibitory activity of leaf oil and leaf oil microcapsules.

Specimen	IC_50_ (μg/mL)
β-CD	—
Cin oil	11.2 ± 0.7 ^b^ **
β-CD/Cin oil	83.3 ± 2.0 ^a^
Allopurinol *	0.5 ± 0.1 ^c^

Cin oil: *C. osmophloeum* leaf oil; β-CD/Cin oil: leaf oil microcapsules; —: no effect; * Positive control; ** Different letters (a–c) in the Table refer to statistically significant difference at the level of *p* < 0.05 according to Scheffe’s test.
